# Novel Method for Osmotic Conductance to Glucose in Peritoneal Dialysis

**DOI:** 10.1016/j.ekir.2020.09.003

**Published:** 2020-09-19

**Authors:** Giedre Martus, Karin Bergling, Ole Simonsen, Eric Goffin, Johann Morelle, Carl M. Öberg

**Affiliations:** 1 Department of Clinical Sciences Lund, Nephrology Division, Skane University Hospital, Lund University, Lund, Sweden; 2 Division of Nephrology, Cliniques Universitaires Saint-Luc, Brussels, Belgium; 3 Institut de Recherche Expérimentale et Clinique, UCLouvain, Brussels, Belgium

**Keywords:** osmotic conductance, osmotic water transport, peritoneal dialysis, peritoneal equilibration test, ultrafiltration

## Abstract

**Introduction:**

The osmotic conductance to glucose (OCG) is a crucial determinant of ultrafiltration (UF) in peritoneal dialysis (PD) patients and can be used to monitor membrane integrity in patients on long-term PD. It has been proposed that OCG can be assessed based on drained volumes in 2 consecutive 1-hour glucose dwells, usually 1.5% and 4.25% glucose, in a so-called double mini-peritoneal equilibration test (dm-PET). However, recent data indicated that the dm-PET provides a poor estimate of OCG unless the residual volume (RV) is taken into account. We introduce an easy, robust, and accurate method to measure OCG and compare it with conventional methods.

**Methods:**

In a prospective cohort of 21 PD patients, a modified version of the dm-PET was performed, along with the determination of RV before, between, and after dwells. Based on computer simulations derived from the 3-pore model (TPM) for membrane permeability, we developed and validated a novel single-dwell method to estimate OCG. We next validated the equation in an independent cohort consisting of 32 PD patients.

**Results:**

Single-dwell OCG correlated more closely with actual UF (*r* = 0.94 vs. r = 0.07 for conventional dm-PET), sodium sieving, and free water transport (FWT) compared with other methods. These findings were replicated in the validation cohort in which OCG calculated using the single-dwell method closely correlated with parameters of osmotic water transport, even when RV was not taken into account, using only drained volumes.

**Conclusion:**

We propose a novel, easy, and robust single-dwell method to determine OCG in individual patients and to monitor membrane integrity over time on PD.

## Graphical abstract

**Figure:**
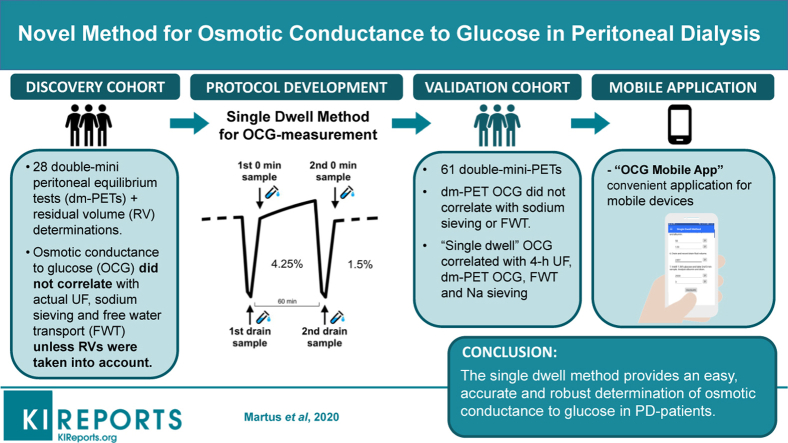

The diagnosis of UF problems in PD patients remains a major challenge. A growing body of evidence shows that overhydration predicts worse survival in dialysis patients,^1^^,^^2^ and data from a large contemporary cohort showed that more than 50% of prevalent PD patients are fluid overloaded.^3^ Water transport across the peritoneal membrane occurs by means of osmosis due to the presence of an osmotic agent in the dialysate, usually glucose. The ability of the membrane to generate UF in response to a crystalloid osmotic gradient can be quantified by OCG. In the clinic, the dm-PET described by La Milia *et al.*^4^ is an increasingly adopted method to estimate OCG in patients (Figure 1a). The difference in milliliters between the drained volume of a 60-minute 1.5% (83.2 mM) glucose dwell followed by a 60-minute 4.25% (239.4 mM) glucose dwell is used to estimate OCG, essentially

$$\text{OCG}\approx \frac{{\text{V}}_{\text{D},4.25}-{\text{V}}_{\text{D},1.5}}{100}\cdot$$

**Figure 1:**
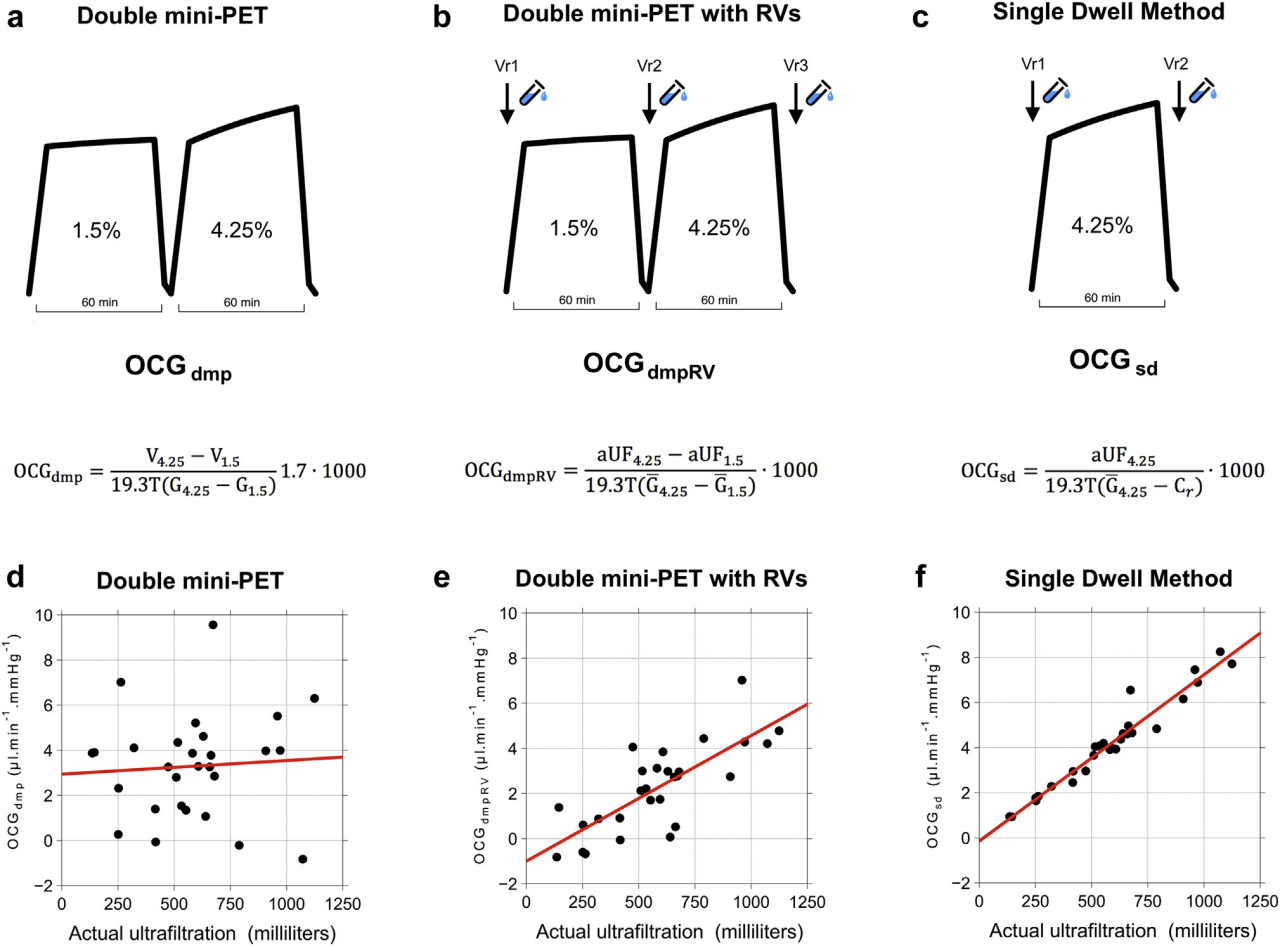
(a) A double mini-peritoneal equilibration test (PET) consisted of 2 consecutive 60 min dwells with 1.5 % glucose followed by 4.25% glucose fluid. Calculation of OCG (OCG_dmp_) is based on stock glucose concentrations (G_4.25_ and G_1.5_) and drained volumes (V_4.25_ and V_1.5_). (b) Modified double mini-PET with residual volumes being assessed before, between, and after the dwells. Calculation of OCG (OCG_dmpRV_) is performed on the basis of actual average glucose concentrations (${\bar{\text{G}}}_{4.25}$ and ${\bar{\text{G}}}_{1.5}$) and actual ultrafiltration (aUF_4.25_ and aUF_1.5_). (c) A single-dwell method to determine OCG, essentially a single mini-PET with residual volume determination before and after a 60-minute 4.25% dwell. Calculation of OCG (OCG_sd_) is based on aUF and actual glucose concentration assuming an opposing osmotic concentration gradient (C_r_) of 40 mmol/L. (d) OCG_dmp_ versus actual 60-minute UF for the 4.25% dwell (aUF_4.25_). (e) OCG_dmpRV_ versus aUF_4.25_. (f) OCG_sd_ versus aUF_4.25_. DPI, dots per inch.

The factor in the denominator will vary depending on the underlying assumptions. Common baseline values for OCG are 3 to 4 μl/min per mm Hg. It has been proposed that a difference of >250 ml between the dwells (i.e., OCG > 2.5) may be regarded as a normal value, whereas a difference <200 ml (i.e., OCG < 2) may indicate the presence of a reduced OCG.^5^ However, the dm-PET is usually performed without consideration of RVs and actual dialysate glucose concentrations, which means that large errors could arise in the estimation of UF (and OCG).^6^^,^^7^

Interestingly, the estimation of OCG has also been performed on the basis of a single dwell as nicely reported by Parikova *et al.*^8^ In the present study, we developed a simplified method to determine OCG based on a single 1-hour 4.25% dwell with RV determination pre- and postexchange and validated the novel method in a discovery cohort and in an independent validation cohort. To facilitate clinical use of the single-dwell methodology, we developed a mobile app.

## Methods

### Patients

In the discovery cohort, 30 dm-PETs were performed in 21 prevalent PD patients at the home dialysis unit at Skåne University Hospital, Lund, Sweden, from September 30, 2015, until October 12, 2017. Prevalent PD patients at the home dialysis unit at Skåne University Hospital in Lund with at least 1 month of PD duration and with no peritonitis during the month preceding the dm-PET were eligible for inclusion. In the validation cohort, 61 peritoneal function tests were performed in 32 PD patients at the Cliniques Universitaires Saint-Luc, Brussels, Belgium. The study was approved by the local ethics committee and is compliant with the Declaration of Helsinki. No adverse events were observed during the duration of the study.

### Modified dm-PET

After informed consent was obtained, patients were scheduled to arrive in the morning to receive 2 L 1.5% glucose. Testing commenced with draining the initial 1.5% and was followed by a 60-minute 1.5% glucose dwell and a 60-minute 4.25% glucose dwell, representing a dm-PET (Figure 1). All bags and connectors were carefully weighed in order to calculate the fill volumes and the drained volumes. Samples were taken from the effluents of the rinse dwell, 1.5% dwell, and 4.25% dwell; 10-ml samples were also obtained from the dialysate immediately after filling the 1.5% dwell and the 4.25% dwell. After draining the 4.25% dwell, the patient received 2 L 1.5% glucose, and a last sample was drawn immediately after instillation.

### Measurements

Measurements of urea, creatinine, Na^+^, and glucose were performed at the Department of Clinical Chemistry, Skåne University Hospital using accredited methods for clinical use (dialysate and serum) using the Cobas 8000/6000 (Roche Diagnostics, Basel, Switzerland). Indirect potentiometry was used for dialysate sodium. Creatinine was measured using an enzymatic method. Dialysate albumin was assessed using immunoturbidimetry using the the Cobas 8000/6000 using the same protocol as that used to determine urinary albumin. The lower limit of detection is 3 mg/l, and the imprecision (coefficient of variation %) is 1.7% at an albumin concentration of 72 mg/l.

### Calculations

The dilution of the albumin concentration in the RV (Alb_1_ assumed equal to that in the drained volume) was measured immediately after filling (Alb_2_) with a known fill volume as follows:

$$\text{RV}=\frac{{\text{Alb}}_{2}}{{\text{Alb}}_{1}-{\text{Alb}}_{2}}\cdot \text{fill volume}\text{.}$$

To validate the RV determination method, we added albumin (0.1 g/l) (Albumin Baxalta; Baxalta Innovations, Vienna, Austria) to known volumes of PD fluid (100–400 ml) and diluted with 2 l fresh PD fluid. The median absolute deviation of RV calculated from albumin dilution from the actual volume was 3.2% and 3.6% for 1.5% and 4.25% fluid, respectively (see Supplementary Figure S1). Knowing the RV before (RV_before_) and after (RV_after_) a dwell allows for a more precise calculation of UF, herein referred to as actual UF (aUF):

$$\text{aUF}=\text{Drained volume}+{\text{RV}}_{\text{after}}-\left(\text{Instilled volume}+{\text{RV}}_{\text{before}}\right)\text{.}$$

Osmotic glucose conductance was calculated (μL · min^−1^ · mm Hg^−1^) from drained volumes using the equation by LaMilia *et al.*^4^ as follows:

$${\text{OCG}}_{\text{dmp}}=\frac{{\text{V}}_{4.25}-{\text{V}}_{1.5}}{19.3\text{T}\left({\text{G}}_{4.25}-{\text{G}}_{1.5}\right)}1.7\cdot 1000\text{.}$$

V_4.25_ and V_1.5_ are the drained volumes for the 4.25% and the 1.5% glucose solutions, respectively. G_1.5_ and G_4.25_ are stock glucose concentrations (mmol/l) for 4.25% and the 1.5% glucose solutions, respectively (reported by the manufacturer). Dwell times T were corrected by adding half of the infusion and drainage time. The corrected dwell times were 60 minutes (interquartile range 57−62 minutes) and 60 minutes (interquartile range 60−61 minutes) for the 1.5% and 4.25% dwells, respectively. When RVs were included, we used the following equation:

$${\text{OCG}}_{\text{dmpRV}}=\frac{{\text{aUF}}_{4.25}-{\text{aUF}}_{1.5}}{19.3\text{T}\left({\bar{\text{G}}}_{4.25}-{\bar{\text{G}}}_{1.5}\right)}\cdot 1000\text{.}$$

aUF_4.25_ and aUF_1.5_ are the actual UFs for the 4.25% and the 1.5% glucose solutions, respectively. ${\bar{\text{G}}}_{4.25}$ and ${\bar{\text{G}}}_{1.5}$ represent the average glucose concentrations during each dwell, assuming a monoexponential dissipation of the dialysate glucose concentration as follows: $\bar{\text{G}}=\left({\text{C}}_{0}-{\text{C}}_{\text{T}}\right)/\text{ln}\frac{{\text{C}}_{0}}{{\text{C}}_{\text{T}}}$, where C_0_ and C_T_ are the measured glucose concentrations at immediately after instillation and immediately before drainage, respectively (see also later).

### Single-Dwell Equation

We recently^9^ derived a simple equation for calculating the UF rate (UFR) during continuous flow PD, namely

$$\text{UFR}=19.3\cdot \text{OCG}\cdot C_{g}-3.1\text{.}$$

However, the counter flow rate of 3.1 ml/min will vary also with the OCG (μL · min^−1^ · mm Hg^−1^), and a more accurate equation when OCG deviates from a normal value is as follows::

$$\text{UFR}=19.3\cdot \text{OCG}\cdot \left(C_{g}-{\text{C}}_{\text{r}}\right)\text{.}$$

C_r_ (mmol/L) is the apparent net average concentration gradient opposing the glucose gradient (mainly consisting of urea, sodium, and glucose), herein estimated to be 40 mmol/l. Assuming a first-order elimination of the dialysate glucose concentration (C_g_), with half-life t_1/2_ = k^−1^ ln 2 from the dialysate, C_g_ can, at any time 0 ≤ *t* ≤ T min, be calculated from ${\text{C}}_{\text{g}}\left(t\right)={\text{C}}_{0}\text{exp}\left(-\text{k}t\right)$where $\text{k}=\frac{1}{\text{T}}\text{ln}\frac{{\text{C}}_{0}}{{\text{C}}_{\text{T}}}$. The amount of UF accumulated between time 0 and T can be obtained as follows:

$$\text{UF}=\overset{\text{T}}{\underset{0}{\int }}19.3\cdot \text{OCG}\cdot \left(C_{g}-{\text{C}}_{\text{r}}\right)\text{ d}t\text{.}$$

Integrating and rearranging gives the following single-dwell equation for OCG:

$${\text{OCG}}_{\text{sd}}=\frac{{\text{aUF}}_{4.25}}{19.3\text{T}\left({\bar{\text{G}}}_{4.25}-{\text{C}}_{\text{r}}\right)}\cdot 1000\text{,}$$

where ${\bar{\text{G}}}_{4.25}=\left({\text{C}}_{0}-{\text{C}}_{\text{T}}\right)/\text{ln}\frac{{\text{C}}_{0}}{{\text{C}}_{\text{T}}}$.

### The 3-Pore Model

We used the TPM^10^^,^^11^ solved with a fourth-order Runge-Kutta method to calculate OCG (${\text{OCG}}_{\text{TPM}}$) for each patient in the cohort. The model was fitted to plasma solute and dialysate concentrations of creatinine, urea, sodium, glucose, and intraperitoneal volume after filling and after 60 minutes of dwell time using a 3-step repeated nonlinear least square regression method^12^ to determine solute diffusion capacities (mass transfer area concentrations [MTACs]) and the membrane UF capacity (LpS). Other model parameters were set according to Supplementary Table S1 in accordance with previous studies.^13^ The ${\text{OCG}}_{\text{TPM}}\;$ was calculated using the following equation:

$${\text{OCG}}_{\text{TPM}}=\left({\text{α}}_{\text{c}}+{\text{α}}_{\text{s}}\cdot {\text{σ}}_{\text{s},\text{g}}+{\text{α}}_{\text{l}}\cdot {\text{σ}}_{\text{l},\text{g}}\right)\cdot \mathrm{LpS}\cdot 1000\text{,}$$

where$\alpha_{c},\alpha_{s}$, and $\alpha_{l}$ are fractional UF coefficients of aquaporins (ultra-small pores), small pores, and large pores, respectively. Diffusion capacities were adjusted to account for the initial vasodilation phenomenon as described by Waniewski *et al.*^14^

### Statistical Analysis

Data are presented as the median (interquartile range) unless otherwise stated. Significant differences were assessed using the Wilcoxon signed rank test. We used an alpha level of 0.05 and a beta level of 0.20 unless otherwise specified. Linear regression was performed using the *lm* function in R (The R Foundation for Statistical Computing, Vienna, Austria). Twenty-eight dm-PETs had adequate statistical power to significantly detect a large effect size (*r* ≥ 0.50) (*pwr* package for R). We used R version 3.5.1 for macOS (The R Foundation for Statistical Computing).

## Results

### Patients and Tests in the Discovery Cohort

A total of 21 prevalent PD patients were prospectively included in the discovery cohort. Baseline and demographic characteristics of the patients are presented in Table 1. The median age of the patients was 70 years; 40% were women, and 95% were white. Twenty-four percent of the patients had diabetes, all had hypertension, and 40% were on automated PD. The aUF (i.e., the net UF corrected for the measured RV) was 153 ml (interquartile range 33–300 ml) for the 1.5% glucose dwell and 588 ml (interquartile range 417–675) for the 4.25% dwell (Table 2). The median sodium removal was 122 mmol/l UF (interquartile range 99–134 mmol/l UF) for the 1.5% glucose dwell and 94 mmol/l UF (interquartile range 81−102 mmol/l UF) for the 4.25% glucose dwell (Table 2).

**Table 1:** Baseline demographics

Characteristic	Value
No. of patients	21
No. of measurements	30
Age, years	75 (70−78)
Sex, *n* (%)	
Male	13 (62)
Female	8 (38)
Ethnicity, *n* (%)	
White	20 (95)
Asian	1 (5)
PD vintage, months (minimum–maximum)	13 (2−78)
PD modality, *n* (%)	
CAPD	13 (62)
APD	8 (38)
BMI, kg/m^2^	25 (23.9–26.1)
Body surface area, m^2^	1.78 (1.73–1.96)
Primary renal disease, *n* (%)	
Glomerulonephritis	5 (24)
CIN	2 (10)
Polycystic kidney disease	3 (14)
Diabetic nephropathy	3 (14)
Hypertensive nephropathy	7 (33)
Amyloidosis	1 (5)
Charlson Comorbidity Index	7 (5−9)
Davies comorbidity index	1 (1−3)
Diabetes, n (%)	5 (24)
Hypertension, n (%)	21 (100)
History of CHF, n (%)	7 (33)
Systolic BP, mm Hg	140 (130−145)
Diastolic BP, mm Hg	83 (80−87)
Plasma concentrations	
Albumin, g/l	33 (31−35)
Glucose, mmol/l	6.8 (6.3−7.4)
Sodium, mmol/l	140 (138−141)
Urea, mmol/l	15 (12−18)

APD, automated peritoneal disease; BMI, body mass index; BP, blood pressure; CAPD, continuous ambulatory peritoneal dialysis; CHF, congestive heart failure; CIN, chronic interstitial nephritis; PD, peritoneal disease.

Data are median (interquartile range) or n (%) unless otherwise specified.

**Table 2:** Parameters of peritoneal transport

Characteristic	Value
Osmotic water transport	
UF 1.5% glucose, ml	153 (33−300)
UF 4.25% glucose, ml	588 (417−675)
Free water transport (4.25%), ml	176 (145−213)
Sodium transport	
NaR 1.5% glucose, mmol/l UF	122 (99−134)
NaR 4.25% glucose, mmol/l UF	94 (81−102)
Dip Na 60 min, mmol/l	9 (8−10)
Osmotic conductance to glucose	
OCG_dmp_, μL · min^−1^ · mm Hg^−1^	3.5 (1.5−4.2)
OCG_dmpRV_, μL · min^−1^ · mm Hg^−1^	4.1 (1.4−5.9)
OCG_sd_, μL · min^−1^ · mm Hg^−1^	4.1 (2.8−4.9)

Dip Na 60, sodium dip at 60 minutes; NaR, sodium removal; OCG, osmotic conductance to glucose; OCG_dmp_, osmotic conductance to glucose calculated from the double mini-peritoneal equilibration test; OCG_dmpRV_, osmotic conductance to glucose calculated from the double mini-peritoneal equilibration test taking residual volumes into account; OCG_sd_, osmotic conductance to glucose calculated using the single-dwell method; OCG_tpm_, osmotic conductance to glucose calculated using the 3-pore model; UF, ultrafiltration calculated taking residual volumes before and after drain into account (see text).

Data are median (interquartile range).

### The Importance of RV in the Determination of dm-PET OCG

**Figure 2:**
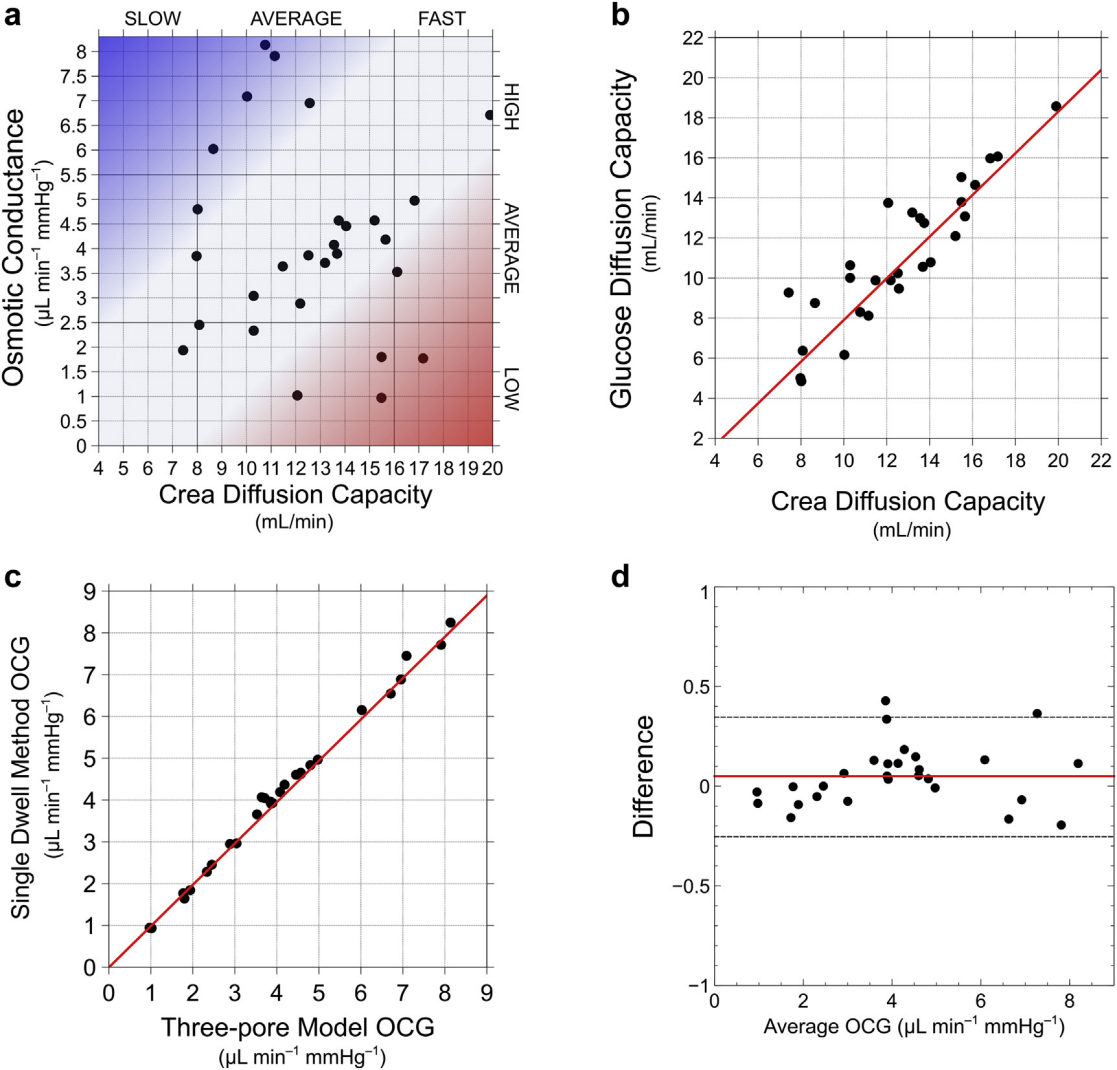
(a) Osmotic conductance to glucose calculated using the 3-pore model (OCG_tpm_) versus creatinine mass transfer area concentration (MTAC_crea_) as estimated with the 3-pore model for all 28 double mini-peritoneal equilibration tests in the discovery cohort. The capability of each individual patient to obtain ultrafiltration (UF) using glucose as an osmotic agent is chiefly dependent on OCG and the small-solute diffusion capacity of the peritoneal membrane, here illustrated by MTAC_crea_. Low glucose conductance and fast transport status → poor UF ability (red color). High glucose conductance and slow transport status → good UF ability (blue color). (b) Glucose diffusion capacity (mL/min) vs creatinine diffusion capacity (ml/min). The ratio between their respective diffusion coefficients (D_glucose_/D_crea_ ≈ 8.8·10^−6^/1.1·10^−5^) is 0.80. (c) Linear regression between OCG estimated with the single- dwell equation (y-axis) and OCG calculated using the 3-pore model (x-axis). (d) The Bland-Altman plot of the difference in OCG between the single-dwell equation and the 3-pore model as a function of the average OCG of the methods.

During a 60-minute dwell with 4.25% glucose, OCG should, by definition, be in a linear relationship with the aUF. In stark contrast, as can be seen in Figure 1d, we found no linear correlation (*r* = 0.07, *P* = 0.73) between the aUF and OCG calculated using the dm-PET equation (OCG_dmp_). Moreover, no correlations were found between OCG_dmp_ and a 60-minute sodium dip or FWT. The aUF and glucose concentrations improved the accuracy of the dm-PET equation, yielding a significant linear correlation between OCG and aUF (*R*^2^ = 57 %, *r* = 0.76, *P* < 0.001) (Figures 1b and 2), a 60-minute sodium dip (*P* < 0.05), and FWT (*P* < 0.01). However, because OCG reflects the UF capacity of both small pores and ultra-small pores, the correlations between OCG and the sodium dip and FWT can only be partial.

### Single-Dwell Method Development and Validation Using the 3-Pore Model

Taking RVs and glucose concentrations into account provided significant correlations between peritoneal water transport and OCG when using the dm-PET method but will also make the procedure more cumbersome and time-consuming. Therefore, on the basis of the TPM for PD, we developed a method to determine OCG on the basis of a single 4.25% dwell (Figure 1c). We assumed that the UF rate at time *t* could be described by the simplified equation

(1) $$\text{UFR}\left(t\right)=19.3\cdot \text{OCG}\cdot (C_{g}\left(t\right)-{\text{C}}_{\text{r}})\text{.}$$

Here *C*_*g*_*(t)* is the dialysate glucose concentration at time *t*, and C_r_ is the apparent opposing net average concentration gradient (estimated to be 40 mmol/L). Next, we modeled an exponential dissipation of the glucose concentration in the dialysate such that *C*_*g*_ is, at any time 0 ≤ *t* ≤ T min, determined by ${\text{C}}_{\text{g}}\left(t\right)={\text{C}}_{\text{g}}\left(0\right)\text{exp}\left(-\text{k}t\right)$, where $\text{k}=\frac{1}{\text{T}}\text{ln}\frac{{\text{C}}_{\text{g}}\left(0\right)}{{\text{C}}_{\text{g}}\left(\text{T}\right)}$. Integration and rearrangement of equation 1 gives

(2) $${\text{OCG}}_{\text{sd}}=\frac{{\text{aUF}}_{4.25}}{19.3\text{T}\left({\bar{\text{G}}}_{4.25}-{\text{C}}_{\text{r}}\right)}\cdot 1000\text{,}$$

where ${\bar{\text{G}}}_{4.25}=\left({\text{C}}_{\text{g}}\left(0\right)-{\text{C}}_{\text{g}}\left(\text{T}\right)\right)/\text{ln}\frac{{\text{C}}_{\text{g}}\left(0\right)}{{\text{C}}_{\text{g}}\left(\text{T}\right)}$. This more simplistic single-dwell equation provided a superior (*R*^2^ = 94%) linear correlation (Figure 2) with aUF. Significant correlations were also found with FWT (*r* = 0.73, *P* < 0.001) and the sodium dip at 60 minutes (*r* = 0.57, *P* < 0.01). The OCG concept is based on the TPM by Rippe.^15^ Accordingly, to validate the single-dwell method, we used the TPM as a reference method in a Bland-Altman analysis. The OCG_TPM_ was estimated in all patients by means of nonlinear regression to determine OCG and the diffusion capacities (MTAC) for glucose, creatinine, and urea. As illustrated in Figure 2c, the linear correlation between the simplified approach and the TPM was excellent (*R*^2^ = 99%, intercept = 7 × 10^−3^, slope = 1.01), validating the single-dwell method. Bland-Altman analysis revealed a small tendency for the equation to overestimate OCG (Figure 2d). The results of the regression analysis for OCG and creatinine MTAC (MTAC_crea_) are displayed in Figure 2a. OCG and MTAC_crea_ were, in line with previous results, not significantly correlated (*r* = −0.03, *P* = 0.86),^4^ suggesting that patients can be independently described as “fast/slow transporters” and “high/low conductors.” These parameters could then, in combination, be used to determine each patient’s ability to obtain UF using glucose-based PD fluids (Figure 2a; red = poor UF ability, blue = good UF ability). We also found a very strong linear relationship between the diffusion capacities (MTAC) of glucose and creatinine (*r* = 0.89) (Figure 2b). The correlation between MTAC_urea_ and MTAC_crea_ was less striking (*r* = 0.69). There was also a moderate correlation between MTAC_urea_ and OCG (*r* = 0.53).

### Diagnostic Accuracy of the Different OCG Methods

The clinical rationale for measuring OCG is to identify patients with defective peritoneal water transport. Using a threshold of 2 μl · min^−1^ · mm Hg^−1^,^5^ the TPM identified 5 patients with low OCG, suggestive of type II UF failure.^16^ The single-dwell method identified these patients while excluding patients not having the condition (Table 3). The dm-PET equation^4^ based on drained volumes detected only 1 of these 5 patients (sensitivity 20%), whereas the specificity was 57%. Considering RV and aUF allowed improving the sensitivity of the dm-PET equation to 100%.

**Table 3:** Diagnostic accuracy of the various methods to determine osmotic conductance to glucose

Index Methods	Reference Method (OCG_tpm_)	
dm-PET, no residual volume	OCG_tpm_ > 2	OCG_tpm_ < 2
OCG_dmp_ > 2	16	4
OCG_dmp_ < 2	7	1
dm-PET, including residual volumes		
OCG_dmpRV_ > 2	16	0
OCG_dmpRV_ < 2	7	5
Single-dwell equation, including residual volumes		
OCG_sd_ > 2	23	0
OCG_sd_ < 2	0	5

dm-PET, double mini-peritoneal equilibration test; OCG_dmp_, osmotic conductance to glucose calculated from the double mini-peritoneal equilibration test; OCG_dmpRV_, osmotic conductance to glucose calculated from the double miniperitoneal equilibration test taking residual volumes into account; OCG_sd_, osmotic conductance to glucose calculated using the single-dwell method; OCG_tpm_, osmotic conductance to glucose calculated using the 3-pore model.

### Performance of the Single-Dwell Method Without RV measurement

To elucidate whether the above findings could be replicated, analysis was performed in a larger cohort of 32 patients in which 61 Uni-PETs (modified PETs which include dm-PETs) were performed as described previously.^7^^,^^17^ Suitable methods for RV measurement may not be available at all clinics, and analysis in the replication cohort was performed without taking the variation of RVs between dwells into account to determine UF (i.e., net UF = drained volume – instilled volume). Baseline and demographic parameters of the cohort are shown in Supplementary Table S2. In this separate analysis, we were able to replicate these findings of significant correlations between OCG_sd_ and FWT (*r* = 0.46, *P* < 0.001) and a sodium dip at 60 minutes (*r* = 0.26, *P* < 0.05) (Table 4). In contrast, both of these parameters describing peritoneal water transport did not correlate with OCG_dmp_. However, there were significant correlations between the 4-hour UF and both OCG_dmp_ (*r* = 0.26, *P* < 0.05) and OCG_sd_ (*r* = 0.44, *P* < 0.01). OCG_sd_ is more closely correlated with 60 minute net UF compared to OCG_dmp_ (Supplemental Figure S2). The replication cohort included a single RV measurement between the 1-hour dwells in the dm-PET, which was moderately correlated to OCG_dmp_ but not to OCG_sd_, indicating the significant influence of the RV on the former parameter.

**Table 4:** Correlation coefficients between parameters of osmotic water transport in a separate cohort not taking residual volumes into account

	Net UF 4 h	Dip Na 60	FWT	OCG_dmp_	OCG_sd_	Residual volume^a^
Net UF 4 h	1.00					
Dip Na 60	0.37^b^	1.00				
FWT	0.42^b^	0.85^b^	1.00			
OCG_dmp_	0.26^c^	0.03	0.19	1.00		
OCG_sd_	0.44^b^	0.26^c^	0.46^d^	0.76^d^	1.00	
Residual volume^a^	0.13	0.06	0.20	0.29^c^	0.11	1.00

FWT, free water transport; Dip Na 60, sodium dip at 60 minutes in mmol/l; OCG_dmp_, osmotic conductance to glucose calculated from the double mini-peritoneal equilibration test; peritoneal equilibration test; OCG_sd_, osmotic conductance to glucose calculated using the single-dwell method; UF, ultrafiltration = drained volume – instilled volume.

a Measured between dwells (see text).

b *P* < 0.01.

c *P* < 0.05.

d *P* < 0.001.

### Single-Dwell OCG Mobile App

To facilitate practical use, we implemented the single-dwell OCG equation into an application for mobile devices. Supplementary Figure S3 shows a screenshot with the link to download and install the application in a mobile device.

## Discussion

Here we developed an easy, robust, and accurate method to determine OCG on the basis of a single 60-minute 4.25% glucose dwell. In line with recent results,^7^ our data confirm that the dm-PET provides a strikingly poor estimate of OCG for individual patients. Our data show that including the variation of RVs between dwells in the dm-PET methodology dramatically improved the correlation with osmotic water transport. Moreover, our results for OCG are in line with those obtained in the seminal study by Parikova and coworkers.^8^ In their study, 70 kDa dextran was applied as an intraperitoneal volume marker to assess the initial (maximal) transcapillary UF rate from which the authors determined OCG on the basis of a single 4.25% glucose dwell. Of interest, in this study, we report similar correlations between OCG and FWT to those found by Parikova and coworkers.^8^ Measuring the RVs in a dm-PET increases the complexity of the procedure, making it more cumbersome and time-consuming. To simplify this procedure, we developed a simplified single-dwell methodology to assess OCG in PD patients. In the absence of 70 kDa dextran administration as performed by Parikova *et al.*,^8^ the main drawback with the method is RV determination, keeping in mind that reliable and sensitive methods to measure albumin or other suitable markers in the effluent may not be available at all clinics. In a separate analysis of 61 tests, we found at least a moderate correlation between OCG and 4-hour UF, even without taking RVs into account. This latter analysis suggests that the method performs better than the dm-PET in individual patients, even when RV is not taken into account. Furthermore, our data show a considerable variability in the RVs between dwells. Although outside the scope of this study, this could be accounted for by 2 main reasons: (i) the position of the peritoneal catheter is not fixed and even small changes of the catheter tip position (1–2 cm) can easily cause large differences in the RV (Supplementary Figure S4) and (ii) incomplete peritoneal effluent drainage.

All methods for clinical OCG determination involve some sort of simplifying assumption to facilitate clinical use. A simplification in the single-dwell method is the assumption of an apparent ∼40 mmol/l average concentration gradient (C_r_) opposing the glucose gradient. Theoretically, this parameter should ideally be set to an individual value for each patient, at least according to the plasma sodium, glucose, and urea concentrations (or the plasma osmolality) of the patient, which make up for most of the apparent opposing gradient, with the oncotic pressure and lymphatic absorption making up only a small portion. Nevertheless, in the current analysis, OCG_sd_ was in good agreement with the more complex OCG_tpm,_ which incorporated these parameters. This may imply that the opposing gradient (mainly the plasma osmolality and oncotic pressure) was similar between patients in this cohort. Also, during a 4.25% glucose dwell, the crystalloid osmotic pressure exerted by dialysate glucose is several-fold (2–3 times) greater than the opposing pressure, and variations in the latter parameter may impart only small variations in OCG. The simplicity of the present procedure facilitates its clinical use because the test may be routinely performed during follow-up visits to the dialysis clinic. Despite the theoretical simplifications made (which we acknowledge may make the method less accurate), it may provide interesting longitudinal data. Another limitation of the present study is the high median age (75 years) of the patients in the discovery cohort, which could reduce the generalizability of our results. In this context, it is of interest to note that similar results were obtained in the verification cohort in which the median age was much lower (i.e., 47 years).

Encapsulating peritoneal sclerosis (EPS) is a rare but serious complication of PD characterized by a severe fibrogenic reaction of the peritoneum.^18^ Although the risk of EPS is strongly associated with the length of time on PD, it was recently shown that EPS risk estimates are lower when calculated using competing risk of death analyses.^19^ Importantly, previous studies have suggested that a reduction in OCG occurs well before the clinical manifestation of EPS^20^ paralleled by an early loss of sodium sieving, a parameter that has been shown to be a strong predictor of EPS.^18^ Thus, an easy and reliable methodology like that described here may allow early EPS recognition and appropriate therapy implementation.

Water and solute removal are important determinants of outcomes for patients treated with PD, and failure to achieve either and/or both of these parameters is a common cause of technique failure and transfer to hemodialysis. On the basis of computer simulations using the TPM of PD, we show that a new methodology based on a single 4.25% glucose dwell provides a superior estimate of OCG compared with previously described methods.

## Disclosure

All the authors declared no competing interests.
