# Supplementary material for: Novel Method for Osmotic Conductance to Glucose in Peritoneal Dialysis
Source: Kidney Int Rep. 2020 Sep 19;5(11):1974–81. doi: 10.1016/j.ekir.2020.09.003 (PMC7610002; doi:10.1016/j.ekir.2020.09.003)
Supplement: Supplementary File (PDF) [file mmc1.pdf]

Method for Osmotic Conductance to Glucose in Peritoneal Dialysis

Giedre Martus[1] M.D., Karin Bergling[1] M.Sc., Ole Simonsen[1] M.D., Eric Goffin[2,3] M.D., Johann Morelle[2,3] M.D. Ph.D., Carl M. Öberg[1] M.D. Ph.D.

[1] *Department of Clinical Sciences Lund, Nephrology Division, Skane University Hospital, Lund University, Lund, Sweden*

[2]*Division of Nephrology, Cliniques universitaires Saint-Luc, Brussels, Belgium;* [3]*Institut de Recherche Expérimentale et Clinique, Université catholique de Louvain, Brussels, Belgium*

SUPPLEMENTAL MATERIAL

Supplemental Table S1 ……………………………………… page 2

Supplemental Table S2 ……………………………………… page 3

Supplemental Figure S1 ……………………………………. page 4

Supplemental Figure S2 ……………………………………. page 5

Supplemental Figure S3 ……………………………………. page 6

Supplemental Figure S4 .... page 7

Modified STROBE-statement ....……………………. page 8

Supplemental Table S1: Three-pore model parameters

Parameter	Value
Small pore radius (r_s) (Å)	43
Large pore radius (r_L) (Å)	250
Fractional small pore UF coefficient (α_S)	0.9
Fractional transcellular UF coefficient (α_c)	0.02
Fractional large pore UF coefficient (α_L)	0.08
Unrestricted pore area over unit diffusion distance for small pores (A_0/DX) (cm)	25,000
Peritoneal lymph flow (L) (ml/min)	0.3
Transperitoneal hydraulic pressure gradient, mmHg	8
Dialysate Temperature (°K)	310
MTAC Sodium, ml/min	5.946
Plasma oncotic pressure, mmHg	22

MTAC, diffusion capacity. Å, 0.1 nm.

Supplemental Table S2: Baseline demographics of Uni-PET cohort.

Characteristic	
**No. of patients**	32
**No. of measurements**	61
**Age at PD-start, years**	47 (35−55)
**Sex, n (%)**	
**Male**	23 (72 %)
**Female**	9 (28 %)
**Ethnicity, n (%)**	
**Caucasian**	27 (84 %)
**Asian**	4 (13 %)
**African**	1 (3 %)
**PD vintage, months (min-max)**	12 (12-48)
**PD modality, n (%)**	
**CAPD**	19 (59%)
**APD**	13 (41%)
**BMI, kg/m^2**	24 (21 – 27)
**Body surface area, m^2**	1.79 (1.66 – 1.91)
**Primary renal disease, n (%)**	
**Glomerulonephritis/sclerosis**	11 (34%)
**Pyelonephritis**	9 (30%)
**Polycystic kidney disease**	2 (5%)
**Hypertensive nephropathy**	1 (3%)
**Diabetic nephropathy**	6 (18%)
**Other**	3 (10%)
**Charlson comorbidity index**	5 (3−6)
**Davies comorbidity index**	1 (0−2)
**Diabetes, n (%)**	9 (30%)
**Hypertension, n (%)**	28 (87%)
**History of CHF, n (%)**	1 (3%)
**Systolic BP, mmHg**	140 (130−150)
**Diastolic BP, mmHg**	85 (80−90)
**Albumin, g/L**	37 (34−39)
**Glucose, mmol/L**	6.8 (6.3−7.4)

Data are median (interquartile range) or n (%) unless otherwise specified.

Supplemental Figure S1: Bland-Altman analysis of RV determination method.

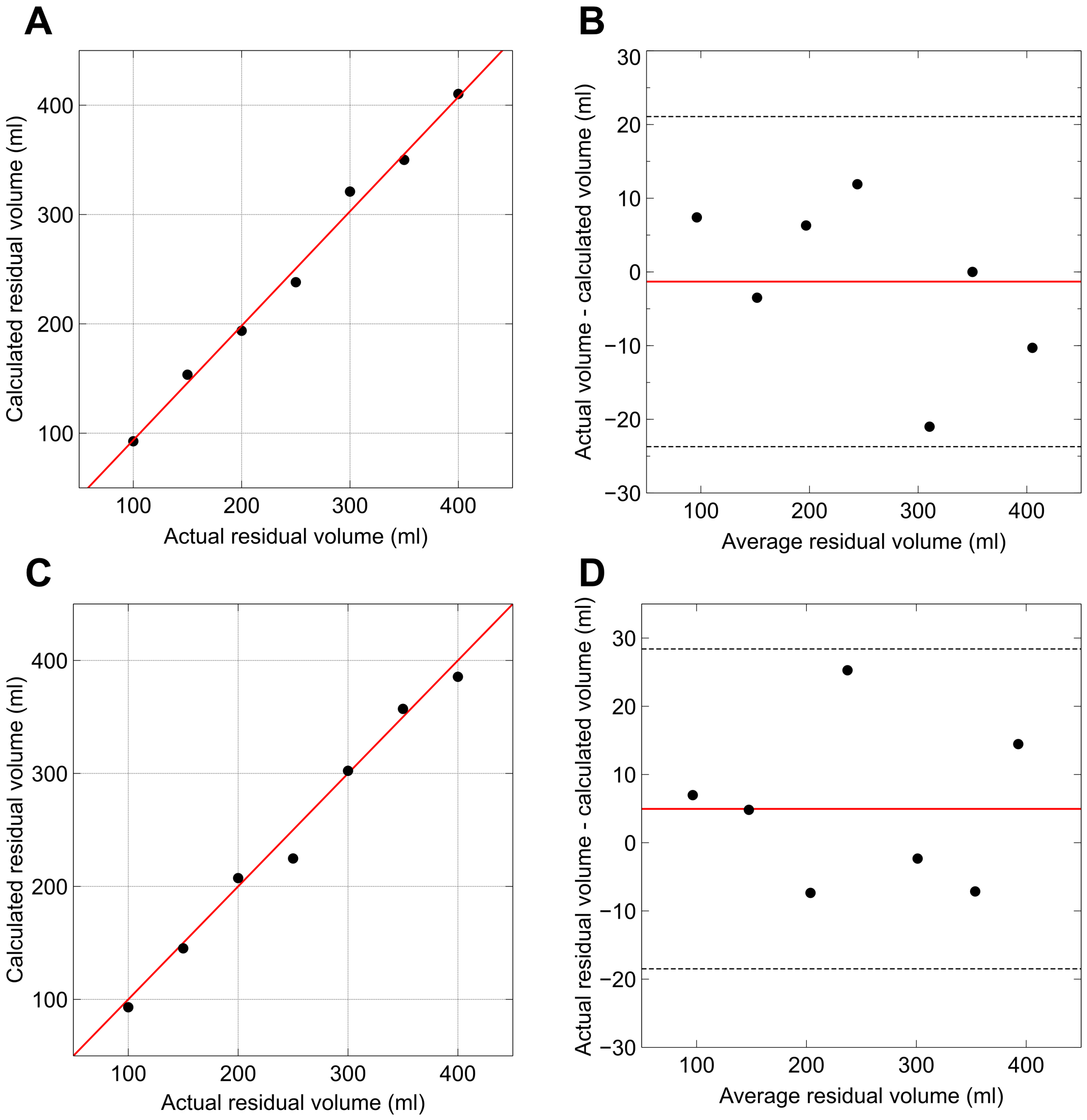

Validation of the RV determination method. Albumin (0.1 g/L) was added to known volumes of PD-fluid (100 mL, 150 mL, 200 mL, 250 mL, 300 mL, 350 mL and 400 mL) and diluted with 2 L fresh PD-fluid. The RV was then calculated from the un-diluted and diluted albumin concentrations. A: Linear regression between calculated and actual volumes for 1.5% fluid and the corresponding difference plot (B). C: Linear regression between calculated and actual volumes for 4.25% fluid and the corresponding difference plot (D).

Supplemental Figure S2: OCG versus 60 min UF without taking RVs into account.

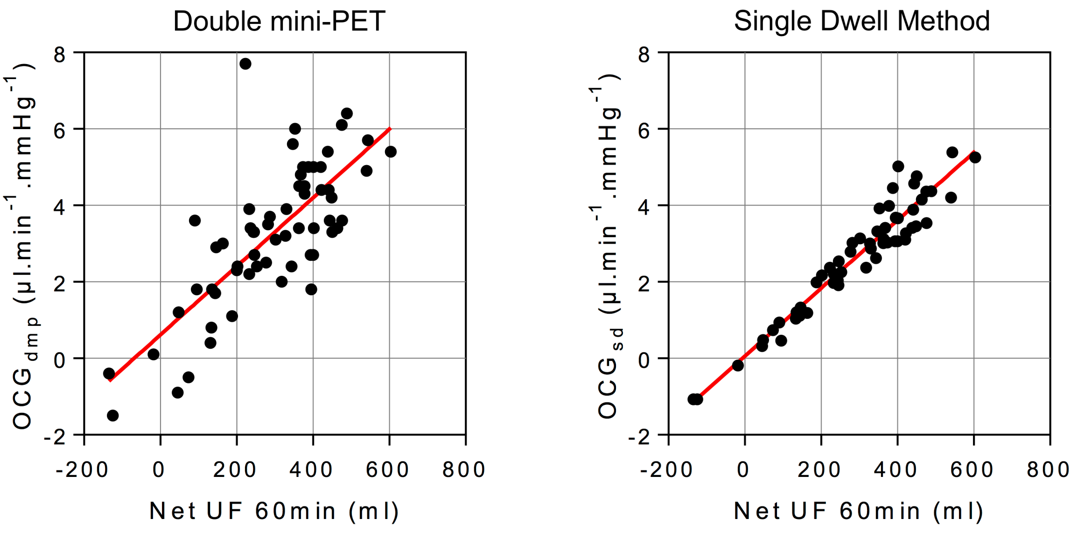

y = 0.01 x + 0.61
R^2=0.58

y = 0.01 x + 0.06
R^2=0.92

Linear correlation between OCG_{dmp} (A) or OCG_{sd} (B) *versus* 60 min UF for 4.25% glucose (UF) without taking the variation of RVs into account, i.e. UF=Drained volume−Instilled volume.

Supplemental Figure 3: Mobile app for OCG calculation.

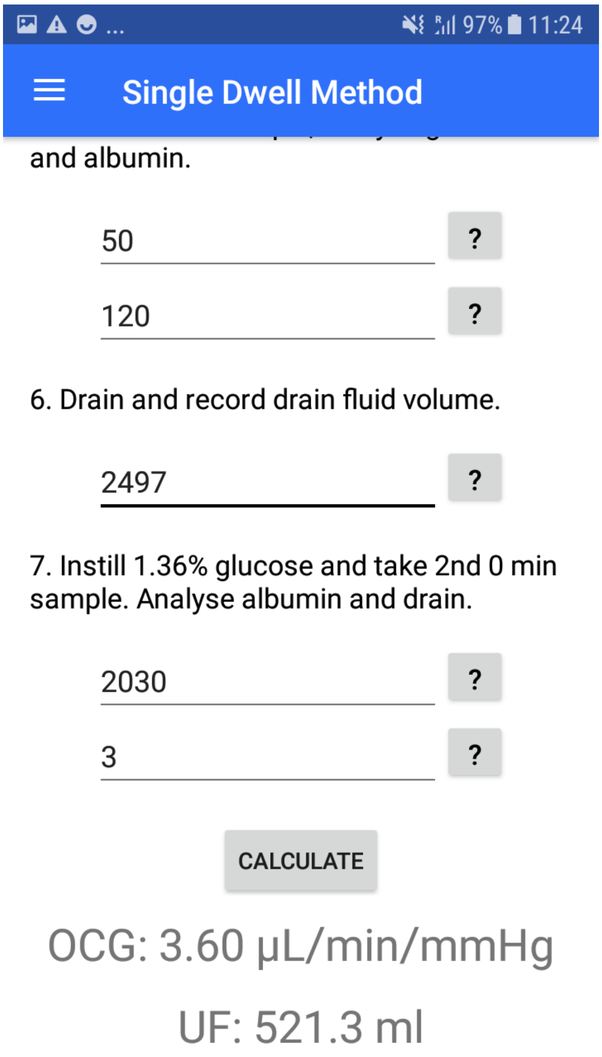

Download link:

https://play.google.com/store/apps/details?id=app.example.test5

Supplemental Figure 4: Influence of catheter tip position on RV.

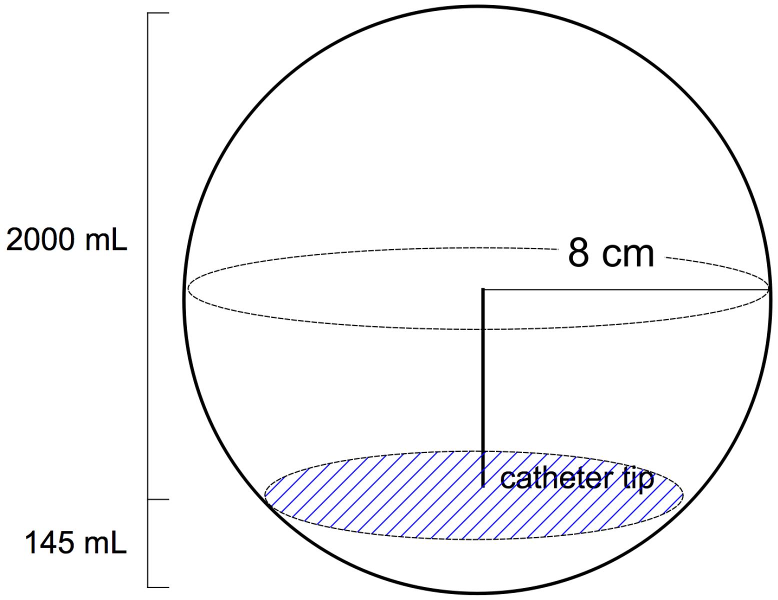

During peritoneal dialysis the intraperitoneal fluid bolus may, as a simplification, be illustrated by a sphere having a radius of 8 cm containing a volume of $\frac{4\pi 8^3}{3} \approx 2145$ mL. Suppose the PD catheter runs along the vertical rotation axis of the sphere so that the tip is located 2.54 cm from the base of the bolus (i.e. leaving a spherical cap containing $\frac{\pi 2.54^2}{3}(3 \cdot 8 - 2.54) \approx 145$ mL). If the tip is moved 1 cm so that it is 3.5 cm from the base, the RV is increased to $\frac{\pi 3.54^2}{3}(3 \cdot 8 - 3.54) \approx 268$ mL (an increment of 85%). If moved 1 cm further, the RV increases even more to 420 mL (+190%).

Modified STROBE Statement—checklist of items that should be included in reports of observational studies (Cohort/Cross-sectional and case-control studies)

	Item No	Recommendation	Page
**Title and abstract**	1	(*a*) Indicate the study's design with a commonly used term in the title or the abstract	2
		(*b*) Provide in the abstract an informative and balanced summary of what was done and what was found	2
**Introduction**			
Background/rationale	2	Explain the scientific background and rationale for the investigation being reported	3, 4
Objectives	3	State specific objectives, including any prespecified hypotheses	3, 4
**Methods**			
Study design	4	Present key elements of study design early in the paper	2-4
Setting	5	Describe the setting, locations, and relevant dates, including periods of recruitment, exposure, follow-up, and data collection	10
Participants	6	(*a*) *Cohort study*—Give the eligibility criteria, and the sources and methods of selection of participants. Describe methods of follow-up<br><br>*Case-control study*—Give the eligibility criteria, and the sources and methods of case ascertainment and control selection. Give the rationale for the choice of cases and controls<br><br>*Cross-sectional study*—Give the eligibility criteria, and the sources and methods of selection of participants	10
Variables	7	Clearly define all outcomes, exposures, predictors, potential confounders, and effect modifiers. Give diagnostic criteria, if applicable	10, 11
Data sources/ measurement	8*	For each variable of interest, give sources of data and details of methods of assessment (measurement).	10, 11
Bias	9	Describe any efforts to address potential sources of bias	4
Study size	10	Explain how the study size was arrived at (if applicable)	14
Quantitative variables	11	Explain how quantitative variables were handled in the analyses. If applicable, describe which groupings were chosen and why	13, 14
Statistical methods	12	(*a*) Describe all statistical methods, including those used to control for confounding	13, 14
		(*b*) Describe any methods used to examine subgroups and interactions	N/A

		(*c*) Explain how missing data were addressed	N/A
		(*d*) *Cohort study*—If applicable, explain how loss to follow-up was addressed<br><br>*Case-control study*—If applicable, explain how matching of cases and controls was addressed<br><br>*Cross-sectional study*—If applicable, describe analytical methods taking account of sampling strategy	N/A
		(*e*) Describe any sensitivity analyses	N/A
**Results**			
Participants	13*	(a) Report numbers of individuals at each stage of study—eg numbers potentially eligible, examined for eligibility, confirmed eligible, included in the study, completing follow-up, and analyzed	7, 10
		(c) **Use of a flow diagram**	N/A
Descriptive data	14*	(a) Give characteristics of study participants (eg demographic, clinical, social) and information on exposures and potential confounders	4
		(b) Indicate number of participants with missing data for each variable of interest	10
		(c) *Cohort study*—Summarise follow-up time (eg, average and total amount)	N/A
Outcome data	15*	*Cohort study*—Report numbers of outcome events or summary measures over time	4
		Case-control study—Report numbers in each exposure category, or summary measures of exposure	
		Cross-sectional study—Report numbers of outcome events or summary measures	
Main results	16	(*a*) Give unadjusted estimates and, if applicable, confounder-adjusted estimates and their precision (eg, 95% confidence interval). Make clear which confounders were adjusted for and why they were included	4-7
Other analyses	17	Report other analyses done—eg analyses of subgroups and interactions, and sensitivity analyses	4-7
**Discussion**			
Key results	18	Summarise key results with reference to study objectives	8

Limitations	19	Discuss limitations of the study, taking into account sources of potential bias or imprecision. Discuss both direction and magnitude of any potential bias	8, 9
Interpretation	20	Give a cautious overall interpretation of results considering objectives, limitations, multiplicity of analyses, results from similar studies, and other relevant evidence	8-10
Generalisability	21	Discuss the generalisability (external validity) of the study results	8

*Give information separately for cases and controls in case-control studies and, if applicable, for exposed and unexposed groups in cohort and cross-sectional studies.

Note: An Explanation and Elaboration article discusses each checklist item and gives methodological background and published examples of transparent reporting. The STROBE checklist is best used in conjunction with this article (freely available on the Web sites of PLoS Medicine at http://www.plosmedicine.org/, Annals of Internal Medicine at http://www.annals.org/, and Epidemiology at http://www.epidem.com/). Information on the STROBE Initiative is available at www.strobe-statement.org.
